# Multi‐generational responses of a marine polychaete to a rapid change in seawater *p* CO_2_

**DOI:** 10.1111/eva.12344

**Published:** 2015-12-18

**Authors:** Araceli Rodríguez‐Romero, Michael D. Jarrold, Gloria Massamba‐N'Siala, John I. Spicer, Piero Calosi

**Affiliations:** ^1^ Departamento de Ecología y Gestión CosteraInstituto de Ciencias Marinas de Andalucía (CSIC)Puerto RealCádizSpain; ^2^ Marine Biology and Ecology Research CentreSchool of Marine Science and EngineeringPlymouth UniversityPlymouthDevonUK; ^3^ College of Marine and Environmental SciencesJames Cook UniversityTownsvilleQldAustralia; ^4^ Dipartimento di Scienze della VitaUniversità di Modena e Reggio EmiliaModenaItaly; ^5^ Département de Biologie Chimie et GéographieUniversité du Québec à RimouskiRimouskiQCCanada

**Keywords:** adaptive potential, climate change, evolutionary adaptation, fecundity, multi‐generational experiment, ocean acidification, parental effects, trans‐generational plasticity

## Abstract

Little is known of the capacity that marine metazoans have to evolve under rapid *p* CO_2_ changes. Consequently, we reared a marine polychaete, *Ophryotrocha labronica*, previously cultured for approximately 33 generations under a low/variable pH regime, under elevated and low *p* CO_2_ for six generations. The strain used was found to be tolerant to elevated *p* CO_2_ conditions. In generations F1 and F2 females’ fecundity was significantly lower in the low *p* CO_2_ treatment. However, from generation F3 onwards there were no differences between *p* CO_2_ treatments, indicating that trans‐generational effects enabled the restoration and maintenance of reproductive output. Whilst the initial fitness recovery was likely driven by trans‐generational plasticity (TGP), the results from reciprocal transplant assays, performed using F7 individuals, made it difficult to disentangle between whether TGP had persisted across multiple generations, or if evolutionary adaptation had occurred. Nonetheless, both are important mechanisms for persistence under climate change. Overall, our study highlights the importance of multi‐generational experiments in more accurately determining marine metazoans’ responses to changes in *p* CO_2_, and strengthens the case for exploring their use in conservation, by creating specific *p* CO_2_ tolerant strains of keystone ecosystem species.

## Introduction

Marine metazoans will face a series of significant changes in oceanic *p*CO_2_ levels over the coming centuries. Firstly, increases in oceanic *p*CO_2_ levels are predicted to occur at an unprecedented rate, leading to a subsequent reduction in pH; a phenomenon commonly termed ocean acidification (OA) (Doney et al. 2009). Secondly, the successful implementation of low‐emissions policies (Frölicher and Joos 2010) and other climate change mitigation measures, such as carbon capture and storage (Bickle 2009; Szulczewski et al. 2012), should result in a decrease of oceanic *p*CO_2_ levels, and thus a rise in seawater pH. Coastal environments will be particularly affected by acidification/‘de‐acidification’ processes due to the unstable equilibrium of coastal water carbonate systems, which are affected by multiple human activities operating at various spatial and temporal scales (Strong et al. 2014). Thus, one of the major challenges in marine global change biology, as well as marine coastal conservation, is to accurately predict how marine metazoans will respond to rapid changes in *p*CO_2_. Whilst the large body of existing literature documents the potential negative impacts of elevated *p*CO_2_ conditions on a wide range of marine species (Kroeker et al. 2013; Wittmann and Pörtner 2013), the majority of studies have focussed on short‐term, within‐generation, effects of modern‐day populations (Kroeker et al. 2013). Consequently, our understanding on the multi‐generational responses of marine metazoans to rapid changes in *p*CO_2_ is limited (Munday et al. 2013; Reusch 2014; Sunday et al. 2014). This lack of knowledge is restricting our ability to accurately predict whether, and how, marine biodiversity will cope with expected environmental *p*CO_2_ changes.

For most marine metazoan species, exposure to changing *p*CO_2_ conditions will occur over multiple generations. Future populations may, therefore, be able to maintain current‐day performance through the process of evolutionary adaptation. Evolutionary adaptation occurs when selection on existing genetic variation shifts the average phenotype of a population towards the fitness peak that matches its present environment (Sunday et al. 2014). A handful of studies have investigated marine metazoan populations naturally exposed to elevated *p*CO_2_ *in situ*, and show that evolutionary adaptation to OA conditions is possible (Maas et al. 2012; Calosi et al. 2013; Lewis et al. 2013; Pespeni et al. 2013). The rate at which such adaptation occurred, however, is unknown. During rapid climate change, evolutionary adaptation will most likely depend on the extent of existing phenotypic/genotypic variation within populations (Lande and Shannon 1996). Several breeding experiments, focussing on early life stages, have shown that sufficient phenotypic/genotypic variation exists within natural populations of marine metazoans, potentially enabling rapid evolutionary adaptation to changes in *p*CO_2_ conditions (e.g. Sunday et al. 2011; Foo et al. 2012; Kelly et al. 2013; Malvezzi et al. 2015). Many marine species, however, have complex life histories, and each life stage can respond to different selection pressures (Miller et al. 2013; Cripps et al. 2014). Therefore, focussing solely on early life stages may over‐ or under‐estimate the potential for any adaptive response to a rapid change in *p*CO_2_ (Sunday et al. 2014).

Nongenetic inheritance mechanisms may also influence the potential for evolutionary adaptation to rapid climatic changes (Chevin et al. 2010; Bonduriansky et al. 2012; Klironomos et al. 2012; Gomez‐Mestre and Jovani 2013). Trans‐generational plasticity (TGP) is the process whereby the environment experienced by parents significantly alters the reactions norms (i.e. phenotypes), and thus fitness, of their offspring (Mousseau and Fox 1998). TGP has the potential to be adaptive, but may also have deleterious effects (Marshall and Uller 2007). Either way, TGP can be an important source of variation in performances between individuals, ultimately influencing short‐term selection and the evolutionary trajectories of populations (Mousseau and Fox 1998; Badyaev and Uller 2009; Bonduriansky et al. 2012). TGP has largely been shown to increase offspring fitness of marine metazoans in response to changes in *p*CO_2_. For example, Miller et al. (2012) showed that preconditioning adult anemone‐fish, *Amphiprion melanopus*, to elevated *p*CO_2_ mediated the negative impacts on juvenile growth, survival and metabolic rates (see also Allan et al. 2014; Murray et al. 2014; Pedersen et al. 2014; Parker et al. 2015). However, the extent to which TGP influences the next generation can depend on whether the parental population has experienced within‐generation acclimation; the process by which an individual alters its physiological, behavioural or morphological characteristics through phenotypic plasticity to better suit an environment (Munday et al. 2013). Recently, Dupont et al. (2013) showed that the pre‐exposure of adult sea urchins to elevated *p*CO_2_ for 4 months had a negative effect on larval survival. In contrast, after 16 months of pre‐exposure, during which adults had acclimated, the negative effect on larval survival was no longer detected (see also Donelson et al. 2012; Suckling et al. 2014). Furthermore, the majority of trans‐generational experiments have been restricted across one, or maximum two, generations leaving it unclear whether or not adaptive TGP can extend into future generations allowing time for evolutionary adaptation to catch up (Chevin et al. 2010), or even facilitate the process (Pigliucci et al. 2006; Crispo 2007; Bonduriansky et al. 2012; Gomez‐Mestre and Jovani 2013).

Breeding and trans‐generational studies to‐date have provided valuable insights into the evolutionary potential of marine metazoans to rapid changes in *p*CO_2_ conditions. However, there is still an urgent need for longer term experiments that encompass all life‐history stages, across multiple generations, to more accurately predict how populations of marine metazoans might respond to the rapid changes in *p*CO_2_ expected to occur (Munday et al. 2013; Sunday et al. 2014). Here, we firstly report the results of a multi‐generational exposure experiment in which we reared a laboratory strain of a marine polychaete, *Ophryotrocha labronica* La Greca and Bacci 1962; for six generations under elevated (1000 μatm) and low (400 μatm) *p*CO_2_ conditions. The *O. labronica* strain used was found to perform better under elevated *p*CO_2_ conditions (generation F1). Secondly, to disentangle whether our observed multi‐generational responses were driven by phenotypic plasticity or by evolutionary adaptation (Calosi et al. 2013; Sunday et al. 2014; Thor and Dupont 2015) we report the results of a reciprocal transplant assay experiment between *p*CO_2_ treatments with F7 individuals. Throughout the experiment we measured a range of fitness‐related life‐history traits, as well as metabolic rates as a proxy for physiological performance. *Ophryotrocha labronica* is a globally distributed benthic polychaete typically found in heterogeneous, organically enriched environments (e.g. fouling communities in harbours, Prevedelli et al. 2005; Simonini et al. 2009). Additionally, it is an excellent species for multi‐generational studies as it can be easily cultured under laboratory conditions and has a short generation time (~17 days at 27°C; Åkesson 1976). It was hypothesized that the multi‐generational exposure to low *p*CO_2_ conditions would enable worms to initially increase, and then maintain, performance levels similar to those observed under elevated *p*CO_2_ conditions. Although we were specifically investigating the response of an elevated *p*CO_2_ tolerant population to low *p*CO_2_ levels, something that is not an immediate conservation issue as compared with OA studies, the results from this study will give valuable insight into how marine metazoans may respond to rapid *p*CO_2_ changes in general.

## Materials and methods

### Animal collection and husbandry

The laboratory strain of *O. labronica* used originated from >40 individuals collected in June 2008 in the harbour of Porto Empedocle (Sicily, Italy; 37°17′4″N, 13°31′3″E). The laboratory strain was housed at the Marine Biology and Ecology laboratory of the University of Modena and Reggio Emilia (Modena, Italy) in culture for approximately 30 generations at relatively constant salinity (mean ± SD: 35 ± 2) (obtained by dissolving an artificial sea salt – Reef Crystals, Instant Ocean – in distilled water) and photoperiod (L:D of 12:12 h), but at variable temperature (min/max = 12/30°C) to mimic natural seasonal variation (Massamba‐N'Siala et al. 2012). In Nov 2012, 120 individuals from a population of approx. 1200 were transported to the Marine Biology and Ecology Research Centre (MBERC) of Plymouth University (Plymouth, UK) and kept for a further three generations at conditions as close as possible to those experienced by the worms just before collection in the laboratory of Modena (*T* = 20 ± 1°C, *S* = 34 ± 1 (0.22 μm filtered natural sea water), L:D of 12:12 h).

### Pre‐experimental phase: pH regimes of the worm cultures

**Figure 1:**
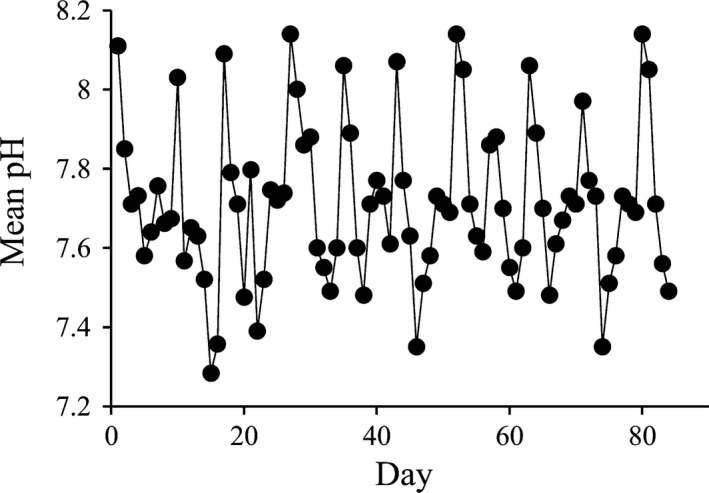
Mean pH of the *Ophryotrocha labronica* worm laboratory cultures measured daily for a 3‐month period.

Initially, we did not have details of the pH regime experienced by the worms in culture for the ~33 generations prior to the start of our experiment. However, our discovery that the strain of *O. labronica* had greater fitness under elevated *p*CO_2_ conditions in generation F1 prompted us to monitor the pH of the culture. Daily measurements for 3 months at MBERC revealed that the worms in culture experienced on average low, but highly variable, pH conditions (Fig. 1; 7.71 ± 0.20) most likely resulting from the feeding and maintenance protocol employed. Briefly, worm cultures were water changed every 7–10 days (the high peaks) and fed on the same day by adding 0.75–1 mL of spinach minced in sea water (300 g L^−1^). The fermentation of spinach during the periods between water changes caused recurrent and persistent reductions in pH (Fig. 1). Although pH data were not collected from the cultures of *O. labronica* at the University of Modena and Reggio Emilia, as the feeding and maintenance protocol of these cultures were identical in the two laboratories we are confident that they too experienced a similar pH regime for the 30 generations in which they were in culture there. In fact, pH measurements of the cultures subsequently made at the University of Modena and Reggio Emilia revealed that pH levels varied depending on how frequently water changes were performed, and ranged from 8.2 to 7.4 (G. Massamba‐N'Siala, personal communication).

### Experimental setup and physico‐chemical parameters

The experimental CO_2_ and temperature manipulation system was a modified version of that described in Pistevos et al. (2011). Briefly, the system comprised of two trays (60 cm × 30 cm × 15 cm, vol. 13 L), half‐filled with de‐ionized water. Each tray housed eight air‐tight experimental chambers (four *per p*CO_2_ treatment). Three chambers *per p*CO_2_ treatment contained a six‐well culture plate (Corning Ltd, Sunderland, UK), filled with filtered natural sea water (0.22 μm, *S* = 33), which contained the worms (three broods of offspring and three adult pairs *per* chamber). The fourth chamber in each treatment contained a glass dish with filtered natural sea water (0.22 μm, *S* = 30) at the same temperature and pH of the wells, which was used for daily partial water changes of the culturing wells. Culture plates were covered with a breathable sealing film (Aeraseal; Alpha Laboratories Ltd, Eastleigh, UK), which allowed gas exchange whilst reducing evaporation, and thus avoiding large salinity and temperature fluctuations.

Elevated *p*CO_2_ conditions were achieved by mixing ambient air, supplied by an aquarium air pump (Mistral 4000; Aqua Medic, Loveland, CO, USA), with CO_2_ gas using adjustable airline gang vales (Algarde; Armitage Pet Care, Nottingham, UK) to produce a nominal *p*CO_2_ concentration of 1000 μatm (year 2100 predicted levels ‐ IPCC, 2013). The CO_2_ content of the resultant gas mixture was measured using a CO_2_ analyser (LI‐840A; Li‐Cor, Lincoln, NE, USA), and then supplied to each chamber *via* an airline connected to a de‐capped micro centrifuge tube (Eppendorf) inserted through the top of the chamber. Low *p*CO_2_ conditions were achieved *via* supplying air with an aquarium air pump (Mistral 4000; Aqua Medic) to each experimental chamber, and represented present‐day levels. The experimental system was maintained at 27°C, as this temperature produced fast generation times (Åkesson 1976) whilst still being within the thermal range naturally experienced in the summer months (Massamba‐N'Siala et al. 2012). The temperature in the water in each tray was controlled by a re‐circulating water bath (R5; Grant Instruments Cambridge Ltd, Herts, UK). Additionally, each tray contained two circulation pumps (Koralia nano 900; Hydor, Sacramento, CA, USA) to ensure an even temperature distribution within the tray (max temperature fluctuation recorded during the experiment was 1°C).

Throughout the experiment, all wells were observed daily using a custom‐built bio‐imaging system for aquatic animals (Tills et al. 2013). Polychaetes were fed daily *ad libitum* on minced spinach (Massamba‐N'Siala et al. 2011, 2012). Uneaten spinach was removed with daily partial water changes to maintain good water quality. Additionally, temperature, salinity and pH were measured daily in one randomly chosen well *per* experimental chamber. Up to twice a week, seawater samples from each treatment were collected to determine total alkalinity. This was done by transferring the water from the glass dish in the fourth experimental chamber of each treatment to a borosilicate bottles (vol. = 150 mL), immediately poisoned with mercuric chloride solution (30 μm, conc. = 0.02%) and kept in the dark prior to analysis. Total alkalinity was measured using an automated acid‐base alkalinity titrating system (AS‐ALK2; Apollo SciTech Inc, Bogart, GA, USA). Carbonate system parameters not measured directly were calculated using CO_2_SYS (Pierrot et al. 2006) and the Mehrbach constants (Mehrbach et al. 1973) refitted by Dickson and Millero (1987). Seawater parameters are presented in Table 1.

**Table 1:** Physico‐chemical parameters of seawater for the elevated and low *p* CO_2_ treatments. Values are means ±1 SD for pH (NBS scale), salinity, temperature, total alkalinity (TA), carbon dioxide partial pressure (*p* CO_2_), bicarbonate and carbonate ion concentration ([${\text{HCO}}_{3}^{-}$]) and ([${\text{CO}}_{3}^{2-}$]), and calcite and aragonite saturation states (Ω_cal_ and Ω_ara_)

Parameter	Elevated	Low
pH	7.68 ± 0.06^A^	7.99 ± 0.06^B^
Salinity	34.05 ± 1.04^A^	33.76 ± 1.24^A^
Temperature (°C)	27.11 ± 0.46^A^	27.16 ± 0.53^A^
TA (μequiv kg^−1^)	2178.77 ± 178.12^A^	2201.66 ± 70.05^A^
*p*CO_2_ (μatm)	1137.28 ± 193.05^A^	461.91 ± 36.21^B^
[${\text{HCO}}_{3}^{-}$] (μmol kg^−1^)	1955.76 ± 167.23^A^	1759.04 ± 58.48^B^
[${\text{CO}}_{3}^{2-}$] (μmol kg^−1^)	92.58 ± 10.76^A^	182.47 ± 14.52^B^
Ω _cal_	2.23 ± 0.26^A^	4.39 ± 0.35^B^
Ω _ara_	1.47 ± 0.17^A^	2.89 ± 0.23^B^

Superscript capital letters indicate a significant difference between treatments by way of GLM's (*P *<* *0.05).

### Experimental design

#### Experiment one: multi‐generational exposure

**Figure 2:**
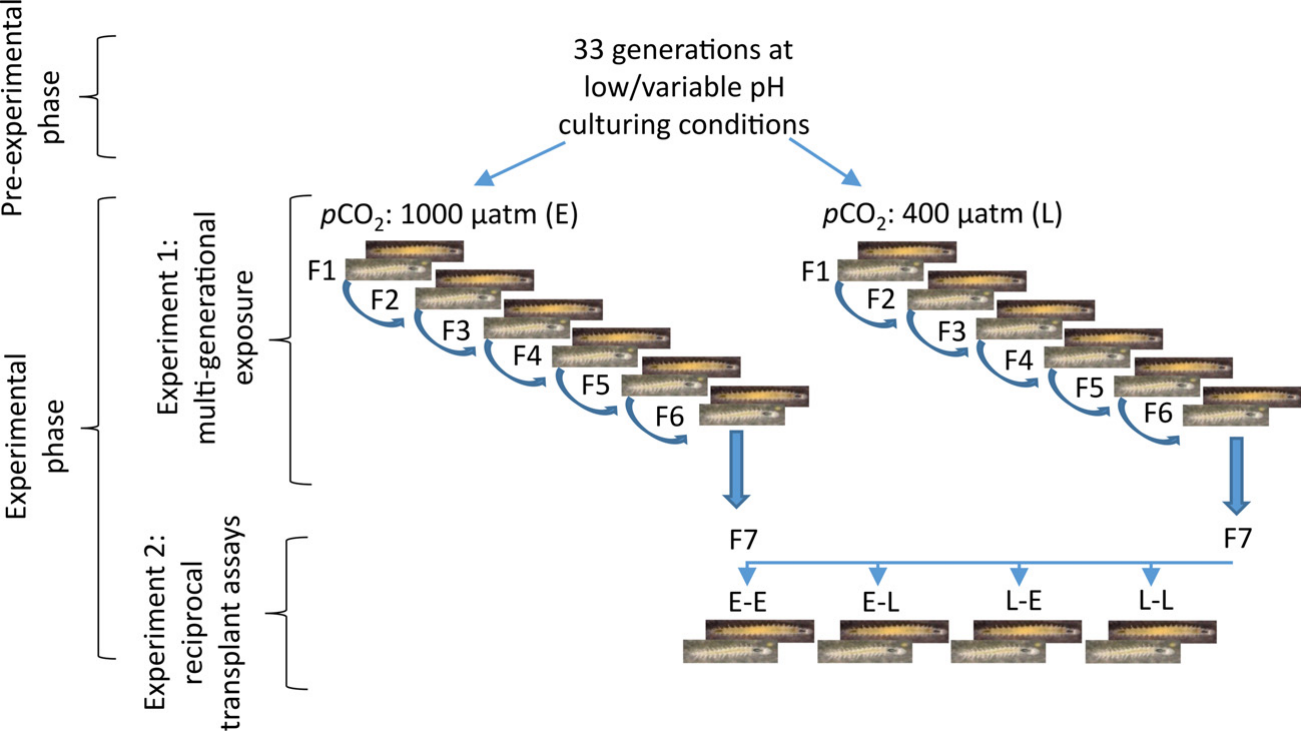
Diagram of the experimental design. E: elevated *p* CO_2_; L: low *p* CO_2_.

To obtain a large enough population of juveniles to conduct the multi‐generational experiment (Fig. 2), 16 breeding pairs were formed from individuals of the MBERC laboratory culture, acclimated (1°C h^−1^) to 27°C. Seventy‐two hours after hatching, 20 juveniles from each pair were haphazardly assigned to either the elevated or low *p*CO_2_ treatment. Juveniles were not moved on the day of hatching as preliminary observation showed that handling of juvenile in the first 72 h from hatching could result in high mortality levels independent of *p*CO_2_ concentrations. Juvenile growth rates and survival were determined 7 days posthatching (see below). When the first reproductive event was observed new pairs were formed by crossing individuals from different parents to avoid inbreeding, and placed into a new well within the same multi‐well plate. Spare worms were kept in their well until offspring from the pairs made were ready to be transferred to a new well and then removed. The first egg mass spawned by a pair was used to produce offspring for the next generation. The second egg mass was used to determine fecundity and egg volume. If a male died before the second egg mass was produced, it was replaced so that the reproductive performance of females could still be determined (as in Massamba‐N'Siala et al. 2011, 2012). For this purpose, spare males of the current generation were kept in the Boveri glass bowl in the fourth experimental chamber of the corresponding treatment. Adult size was determined the day that a female produced its second egg mass. The day after a pair had produced their second egg mass, metabolic rates of the female were measured and the male was removed. The above procedure was repeated up until the stage where generation F6 reached maturity. Adult life‐history traits, as well as metabolic rates, were only measured in females because their contribution to life‐history depiction is more relevant than that of males (Stearns 1992; Massamba‐N'Siala et al. 2011).

#### Experiment two: reciprocal transplant assays

Once F6 juveniles reached maturity, five females *per* brood were moved into a separate well until they reached a sufficiently large size to ensure enough offspring could be produced in their first brood to perform reciprocal transplants between *p*CO_2_ treatments. Consequently, data were not collected for generation F6 adults. Females were then paired up with males from different parents as in all previous generations. From each pair, 20 juveniles were moved to a different well in the same *p*CO_2_ treatment as their parents (control assay) and 20 juveniles were moved to a well in the other *p*CO_2_ treatment (reciprocal assay) 72 h posthatching (Fig. 2). The experimental procedure followed was identical to that described for generations F2–F5, except that the first three egg masses spawned by a pair were used to determine fecundity, and that metabolic rates of females were measured the day after they spawned their third egg mass and were only carried out haphazardly on half of the females.

### Determination of life‐history traits

Juvenile growth rates were determined by counting the number of chaetigers (i.e. segments bearing bristles) of five randomly selected individuals *per* brood using the bio‐imaging system. Juvenile growth rates were standardized to the number of chaetigers added *per* day (number of chaetigers day^−1^).

Juvenile survival *per* well was measured by counting the number of individuals in a well under low power magnification (×10) (MZ12; Leica, Solms, Germany), and was expressed as the percentage of the total number of individuals at time zero.

Female fecundity was measured as the number of eggs produced *per* chaetiger (number of eggs chaetiger^−1^) to account for any effect due to differences in body size (Massamba‐N'Siala et al. 2011, 2012). Counts were made under low‐medium magnification (×50) (MZ12; Leica).

Adult size was determined using the bio‐imaging system by counting the number of chaetigers a female had on the day it produced its second egg mass.

Egg volume, a proxy for egg quality (Allen and Marshall 2014), was determined by taking a picture of the second egg mass using the bio‐imaging system. The longest and shortest axes of 10 eggs were then measured using imageJ (http://rsb.info.nih.gov/ij/) and egg volume (expressed as × 10^−3^ mm^3^) calculated using the formula:

$$V=\frac{4}{3}\pi A^{2}B,$$

where *A* is the short radius and *B* the long radius (Simonini and Prevedelli 2003).

### Determination of metabolic rates

Oxygen uptake was measured as a proxy for metabolic rate (MO_2_) using closed system respirometry. The partial pressure of oxygen (*p*O_2_) in sea water within the respirometry chambers was determined using a modified version of the method described by Calosi et al. (2013) for small‐size worms. Briefly, polychaetes were placed individually into a respirometry chamber (vol. = 0.12 mL), filled with filtered sea water (0.22 μm, *S* = 33–34) of the original *p*CO_2_ treatment. Once sealed, the chambers were placed inside a water bath to maintain constant temperature (*T* = 27 ± 0.5°C). All individuals were allowed to settle for 30 min before measurements began. The decline in *p*O_2_ within each respirometer was determined using an optical oxygen analyser system (GEN III 5000 series; OxySense, Dallas, TX, USA). Measurements of *p*O_2_ were made at regular intervals (20–30 min) for every respirometer for a period of approx. 1.5 h, and the *p*O_2_ in the respirometer never fell below 70% O_2_ saturation to avoid polychaetes experiencing hypoxia. MO_2_ was calculated as the change in *p*O_2_ h^−1^ from the linear least‐squares regression of *p*O_2_ (mbar) plotted against time (min). This was multiplied by the solubility coefficient for oxygen, adjusted for salinity and temperature (Green and Carrit 1967), and the volume of water within each respirometer. Blanks were also run to correct for any microbial respiration in the sea water. MO_2_ values were expressed as μmol O_2_ h^−1^ STPD.

### Statistical analysis

The effects of ‘*p*CO_2_ treatment’, ‘generation’ and their interaction (experiment one) on all traits was tested using general linear models (GLM's), with ‘tray’ as a random factor nested within ‘*p*CO_2_ treatment’, ‘tub’ as a random factor nested within ‘*p*CO_2_ treatment’ × ‘tray’ and ‘well’ as a random factor nested within ‘*p*CO_2_ treatment’ × ‘tray’ × ‘tub’ (juvenile growth rates and egg volume only). Additionally, body size was used as a covariate for MO_2_ data. Random, covariate factors and interactions that had no significant effect (*P *>* *0.05) were systematically removed one at a time (highest *P* value first) from the analysis, until only significant factors and the main effects were left. ‘Well’ had a significant effect on juvenile growth rates (*F*_34, 1097_ = 2.71, *P *<* *0.001). ‘Tub’ had a significant effect on juvenile survival, adult size, metabolic rates and egg volume (min *F*_10,170_ = 2.09, *P *=* *0.028). However, removing these factors from the analysis did not change the patterns of significance of the main factors, and thus effects were considered marginal. Data from the control transplants of the reciprocal transplant assay experiment in generation F7 were included in the analyses. The effect of reciprocal transplant assays (experiment two) on all traits was analysed using the same design, but with ‘exposure treatment’ and ‘assay treatment’ set as fixed factors.

Except for fecundity and metabolic rate data (max. KS_36_ = 0.106, *P *=* *0.200, Kolmogorov–Smirnov's test), data did not meet the assumptions of normality despite Log_10_ transformations (min. KS_229_ = 0.144, *P *<* *0.001). Variances were homogenous for adult size in experiment one and for all data in experiment two (max. *F*_3,67_ = 2.36*, P* = 0.079, Levene's test), except egg volume (*F*_3,706_ = 3.42, *P* = 0.017). For all other datasets assumptions were not met following Log_10_ transformations (min. *F*_11,2077_ = 2.45*, P* = 0.005). However, given the size of our experiment and replication, we assumed the GLM design employed should be tolerant to deviation from the assumptions of normality and heteroscedasticity (Sokal and Rohlf 1995; Underwood 1997). Nonetheless, we also tested the residuals from each analysis against the factors tested with GLM's and no significant relationships were detected (*P *>* *0.05). All pair‐wise comparisons were conducted using 95% confidence interval levels (95% CI). All analyses were conducted using SPSS 21 (IBM, Armonk, NY, USA).

## Results

### Experiment one: multi‐generational exposure

**Figure 3:**
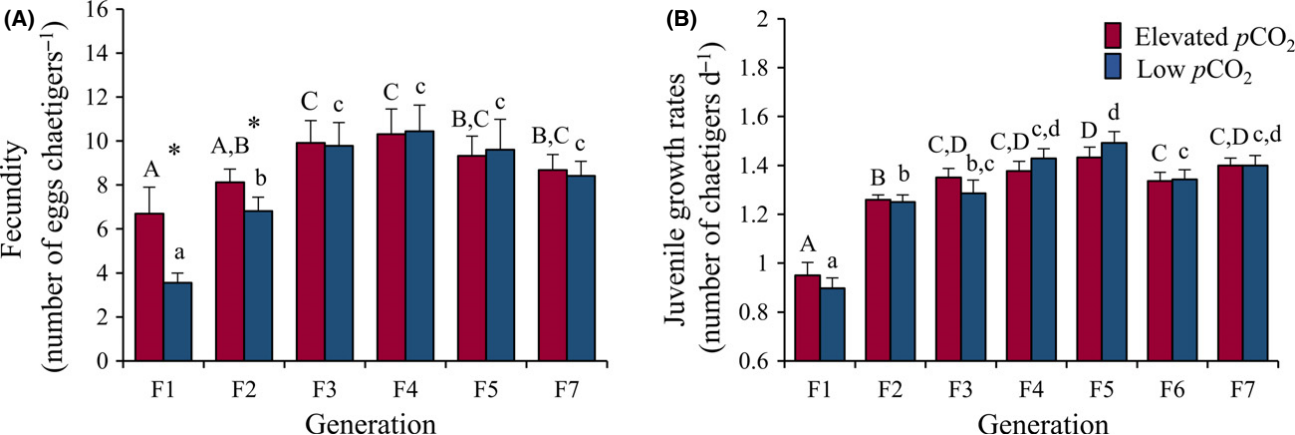
Experiment 1: effect of the multi‐generational exposure to elevated and low *p* CO_2_ on mean fecundity (A) and juvenile growth rates (B) of *O. labronica*. Asterisk (*) indicates significant differences (*P *<* *0.05) between *p* CO_2_ treatments within a generation. Capital and lower case letters represent significant differences (*P *<* *0.05) between generations for the elevated and low *p* CO_2_ treatments, respectively. Bar charts represent mean values ±95% CI. Numbers of replicates are reported in Table S1.

All data produced from the multi‐generational exposure experiment are presented in Table S1. The only trait significantly affected by multi‐generational exposure to low *p*CO_2_ was fecundity (Fig. 3A; *F*_5,197_ = 3.53, *P* = 0.004). In generation F1, mean fecundity in the elevated *p*CO_2_ treatment (6.70 eggs chaetiger^−1^) was significantly greater than in the low *p*CO_2_ treatment (3.55 eggs chaetiger^−1^; *P *<* *0.05). Similarly, in generation F2 fecundity was significantly greater in the elevated *p*CO_2_ treatment (8.12 eggs chaetiger^−1^) compared to the low *p*CO_2_ treatment (6.82 eggs chaetiger^−1^; *P *<* *0.05). However, this difference did not re‐appear in subsequent generations (Fig. 3A; *P *>* *0.05).

For all traits, except egg volume (*F*_5,2078_ = 0.25, *P* = 0.942), a significant generation effect was observed (Table 2; min. *F*_5,180_ = 2.67, *P* = 0.023). Mean juvenile growth rates, fecundity and adult size all significantly increased across generations. For all traits, mean values in generation F1 were significantly lower compared to generation F7 (*P *<* *0.05). However, despite a significant generation effect in juvenile survival and metabolic rate data, no clear trends were present; for both these traits, generation F1 values being statistically similar to generation F7 (*P *>* *0.05). For an explanation on observed significant generation effects please refer to Supporting information.

**Table 2:** Mean generation values ±95% CI for all traits measured for the marine polychaete *Ophryotrocha labronica*

Trait	Generation						
	F1	F2	F3	F4	F5	F6	F7
Juvenile growth rates (number of chaetigers day^−1^)	0.92 ± 0.04^A^ (80)	1.25 ± 0.02^B^ (180)	1.31 ± 0.03^C^ (175)	1.41 ± 0.03^E^ (180)	1.46 ± 0.03^E^ (170)	1.34 ± 0.03^C,D^ (180)	1.40 ± 0.03^D,E^ (180)
Juvenile survival (%)	79.69 ± 10.10^A,B^ (16)	83.33 ± 3.78^A,B^ (36)	80.29 ± 4.96^A^ (35)	87.22 ± 4.29^A,B^ (36)	89.71 ± 3.51^B^ (34)	88.61 ± 2.80^B^ (36)	83.47 ± 2.46^A,B^ (36)
Adult size (number of chaetigers)	14.21 ± 0.34^A^ (34)	15.37 ± 0.30^B^ (35)	15.44 ± 0.28^B^ (34)	15.89 ± 0.21^B^ (35)	15.75 ± 0.33^B^ (36)		15.40 ± 0.22^B^ (35)
Fecundity (number of eggs chaetiger^−1^)	5.13 ± 0.83^A^ (34)	7.45 ± 0.48^B^ (35)	9.85 ± 0.72^C,D^ (34)	10.38 ± 0.81^D^ (35)	9.46 ± 0.82^C,D^ (36)		8.55 ± 0.48^C^ (35)
Egg volume (×10^−3^ mm^3^)	0.61 ± 0.01 (340)	0.62 ± 0.01 (350)	0.62 ± 0.01 (340)	0.61 ± 0.01 (350)	0.61 ± 0.01 (360)		0.61 ± 0.01 (350)
Metabolic rates (μmol O_2_ h^−1^ STPD)	0.23 ± 0.02^A^ (34)	0.27 ± 0.03^A,B^ (35)	0.27 ± 0.02^A,B^ (34)	0.27 ± 0.02^A,B^ (35)	0.29 ± 0.02^B^ (36)		0.29 ± 0.04^A,B^ (18)

Capital letters represent significant differences (*P *<* *0.05) between generations. Numbers of replicates are provided in parentheses.

Finally, for mean fecundity and juvenile growth rates there was a significant difference in the rate of increase between *p*CO_2_ treatments across generations (Fig. 3A,B). Although maximum mean fecundity and juvenile growth rates values were statistically similar for both *p*CO_2_ treatments (*P *>* *0.05), they were reached a generation faster in the elevated *p*CO_2_ treatment. In the elevated *p*CO_2_ treatment, maximum mean fecundity values and juvenile growth rates were reached in generation F2 and F3, respectively, whereas in the low *p*CO_2_ treatment, they were reached in generation F3 and F4 respectively. These differences in rate of increase are highlighted by significant *p*CO_2_ × generation interactions (min. *F*_6,1131_ = 2.93, *P* = 0.008).

### Experiment two: reciprocal transplant assays

**Figure 4:**
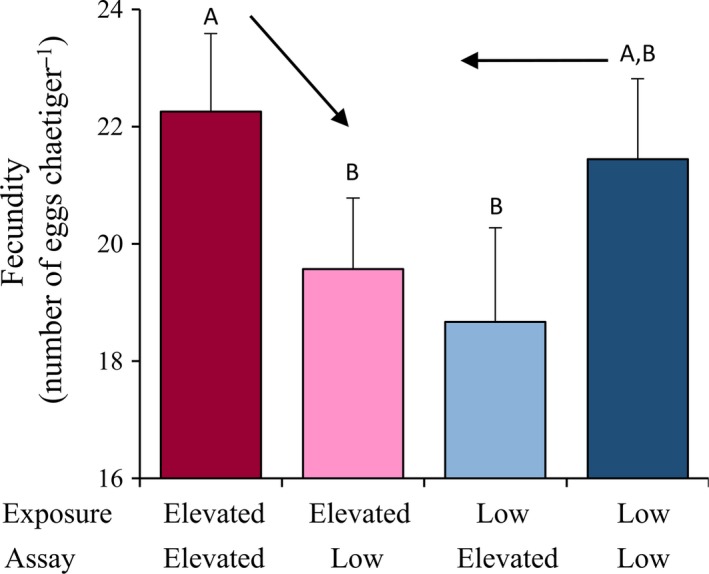
Experiment 2: reciprocal transplant assays. Mean fecundity of *O. labronica* individuals of the F7 generation exposed to either an elevated or low *p* CO_2_ and then transplanted to same (control assay) or other *p* CO_2_ treatments (reciprocal assay). Capital letters represent significant differences (*P *<* *0.05) between transplant treatments. Bar charts represent mean values ±95% CI. Arrows represent reaction norms between control and reciprocal transplants. Numbers of replicates are reported in Table 3.

All data are presented by *p*CO_2_ treatment in Table 3. As in experiment one, fecundity was the only trait that was significantly affected by reciprocal transplants between *p*CO_2_ treatments indicated by a significant exposure × assay interaction (Fig. 4; *F*_1,66_ = 14.83, *P *<* *0.001). Mean fecundity was 22.25 eggs chaetiger^−1^ in the elevated‐elevated *p*CO_2_ transplant and was significantly greater than the elevated‐low and low‐elevated *p*CO_2_ cross (*P *<* *0.05), where mean fecundity was 19.54 and 18.67 eggs chaetiger^−1^, respectively. However, there was no significant difference in mean fecundity between the low‐low *p*CO_2_ transplant (21.44 eggs chaetiger^−1^) and low‐elevated *p*CO_2_ transplant (18.66 eggs chaetiger^−1^) (*P *>* *0.05). There were also no significant differences in fecundity between respective control (i.e. elevated‐elevated versus low‐low) and reciprocal (elevated‐low versus low‐elevated) transplant assays (*P *>* *0.05 in each case).

**Table 3:** Mean values ± 95% CI for all traits measured in the marine polychaete *O. labronica* in the reciprocal transplant assay experiment

Trait	Transplant assay			
	Elevated‐Elevated	Elevated‐Low	Low‐Low	Low‐Elevated
Juvenile growth rates (number of chaetigers day^−1^)	1.40 ± 0.03 (90)	1.40 ± 0.04 (85)	1.40 ± 0.04 (90)	1.39 ± 0.04 (90)
Juvenile survival (%)	82.22 ± 4.09 (18)	78.23 ± 4.59 (17)	84.72 ± 2.91 (18)	79.44 ± 5.43 (18)
Adult size (number of chaetigers)	15.41 ± 0.29 (17)	15.12 ± 0.33 (17)	15.39 ± 0.32 (18)	14.94 ± 0.24 (18)
Fecundity (number of eggs chaetiger^−1^)	22.25 ± 1.33^A^ (17)	19.54 ± 1.21^B^ (17)	21.44 ± 1.38^A,B^ (18)	18.67 ± 1.61^B^ (18)
Egg volume (×10^−3^ mm^3^)	0.62 ± 0.02 (170)	0.59 ± 0.02 (170)	0.60 ± 0.02 (180)	0.60 ± 0.02 (180)
Metabolic rates (μmol O_2_ h^−1^ STPD)	0.28 ± 0.05 (9)	0.31 ± 0.03 (9)	0.29 ± 0.06 (9)	0.27 ± 0.03 (9)

Capital letters represent significant differences (*P *<* *0.05) between treatments. Numbers of replicates are provided in parentheses.

## Discussion

The *O. labronica* strain used in our study had acquired tolerance to elevated *p*CO_2_ during the pre‐experimental phase, through multiple generations of culturing under low/variable pH conditions in the laboratory. Individuals exhibited greater fitness (i.e. fecundity) under elevated *p*CO_2_ (generations F1 and F2). However, we show that between generations F1 and F3 worms were able to increase their fitness under low *p*CO_2_ conditions to levels similar to those observed in the elevated *p*CO_2_ treatment, and that this was likely driven by TGP. From generation F3, worms were able to maintain their restored fitness levels for multiple generations (F3–F7). Hereafter, we discuss the consequences of long‐term culture under a low and variable pH regime, the initial TGP response and the consequences of multi‐generational exposure to low *p*CO_2_ conditions.

### Consequences of laboratory culture under a low and variable pH regime

The worms used in this study exhibited significantly reduced fitness (i.e. fecundity) when initially exposed to low *p*CO_2_ conditions (generations F1 and F2). However, we cannot fully discount that our observed tolerance to elevated *p*CO_2_ conditions was driven by pre‐experimental conditions in the laboratory, other than pH, as we did not have access to worms that had been maintained in culture under stable high pH conditions. Nonetheless, long‐term laboratory culture resulted in worms that performed better under elevated *p*CO_2_ conditions. Additionally, we found that adult size and metabolic rates were unaffected by exposure to low *p*CO_2_. Whilst this shows that individuals were able to acclimate morphologically and physiologically to low *p*CO_2_ conditions, it suggests a change in energy allocation (Stumpp et al. 2011; Fitzer et al. 2012; De Wit et al. 2015; Thor and Dupont 2015) away from reproduction to maintain growth and cellular homeostasis. This shift in energy allocation is consistent with life‐history theory for invertebrates where growth continues after maturation, such as *O. labronica*, as investment into growth increases future reproductive performance; fecundity being related to female size (Reznick 1983; Berglund 1991). *Ophryotrocha labronica* females can allocate up to 84% of available energy into reproduction under favourable conditions (Cassai and Prevedelli 1999). The reduced reproductive output under low *p*CO_2_ conditions is therefore likely due to a portion of this energy being used to support an increased energetic cost of living in a stressful environment, possibly related to disruptions in acid‐base regulation and ion transport (Pörtner et al. 2004; Pörtner 2008). Indeed, physiological alterations caused by acclimation (i.e. phenotypic plasticity) are often associated with fitness costs/life‐history trade‐offs (Hoffmann 1995; Angilletta et al. 2003).

### The initial TGP response

Although fecundity was reduced in individuals exposed to low *p*CO_2_ conditions in both generations F1 and F2, the magnitude of this difference was reduced across generations (F1 = 47.0% vs. F2 = 16.0%). Furthermore, from generation F3 onwards there was no longer a significant difference in fecundity between treatments, suggesting that routine energy allocation had been restored. In contrast, the negative effects of elevated *p*CO_2_ on larval development and naupliar production in the copepod *Tisbe battagliai* remained after multi‐generational exposure (Fitzer et al. 2012). It is possible that evolutionary adaptation could have occurred after only two generations (e.g. Christie et al. 2012), however, we argue that TGP was likely the underlying mechanism for our observed restoration in fecundity. In support of this suggestion, we observed no significant differences in juvenile survival between the two *p*CO_2_ treatments, from which we inferred that no evolutionary adaptation, *via* natural selection, had occurred.

Grandparental, in addition to parental, environment may have influenced the performance of individuals from generation F3. This was evidenced by the full restoration of fecundity requiring two generations of exposure to low *p*CO_2_. The influence TGP may have on the next generation can depend on whether the parental population has experienced within‐generation acclimation (Donelson et al. 2012; Dupont et al. 2013; Suckling et al. 2014), as previously discussed. Within‐generation acclimation can refer to both nonpermanent responses (i.e. reversible acclimation), that occur when an organism is exposed to short‐term environmental fluctuations, and permanent, irreversible responses that are mainly established during early ontogeny (i.e. developmental acclimation) (West‐Eberhard 2003; Angilletta 2009). *Ophryotrocha labronica* undergoes direct development. In generation F1, offspring were initially moved to low *p*CO_2_ conditions 3 days posthatching. As a consequence, juvenile worms may have experienced limited developmental acclimation. Therefore, it is possible that the acclimation to low *p*CO_2_ conditions we observed in generation F1 was reversible, and consequently not sufficient for TGP to be fully expressed across one generation. Phenotypic plasticity may take at least two generations to be fully expressed, as seen in this study, due to the cumulative effects of different forms of acclimation (e.g. developmental and trans‐generational) (Munday et al. 2013).

#### Potential mechanism for the observed TGP

Trans‐generational plasticity can occur through a variety of mechanisms, including the transfer of nutritional (e.g. maternal provisioning) and molecular (e.g. epigenetic) material (Bonduriansky et al. 2012). In this study, there was no difference in egg volume (a proxy for egg quality) between *p*CO_2_ treatments in any generation, indicating no apparent differences in maternal provisioning (see also Miller et al. 2012; Shama et al. 2014). Trans‐generational epigenetic effects (Jablonka and Raz 2009; Ho and Burggren 2010) may therefore have been the causative mechanism for restoring and maintaining routine energy allocation, and subsequently fecundity, under low *p*CO_2_ conditions. Trans‐generational epigenetic effects were also thought to be responsible for mediating the negative impacts that elevated *p*CO_2_ had on juvenile anemonefish, *A. melanopus* (Miller et al. 2012). The extent to which epigenetic effects are induced, is suggested to be dependent upon which life‐stage experiences the stressor, being greater in earlier life stages (Burton and Metcalfe 2014). Consequently, the fact that offspring were initially exposed to low *p*CO_2_ in generation F1, 3 days posthatching, may have meant that epigenetic effects were not fully induced. This again could potentially explain why it took two generations for our observed TGP to be fully expressed. Irrespective of the mechanism involved, our study adds to the growing body of evidence showing that TGP can be an effective mechanism in buffering populations of marine metazoans against the negative effects of changes in *p*CO_2_ and other climate change stressors (e.g. Jensen et al. 2014; Munday 2014; Pedersen et al. 2014; Parker et al. 2015).

### Consequences of multi‐generational exposure to low *p*CO_2_ conditions

#### Reciprocal transplant assay experiment: TGP or evolutionary adaptation?

A reciprocal transplant assay experiment was performed in order to determine whether TGP had persisted across several generations, or if evolutionary adaptation had occurred (Calosi et al. 2013; Sunday et al. 2014; Thor and Dupont 2015). Fecundity levels were significantly lower in individuals transplanted from low to elevated *p*CO_2_ (low‐elevated) compared to those individuals from the elevated *p*CO_2_ control line (elevated‐elevated). This change in reaction norm of the experimental lines lends support to the idea that evolutionary adaptation to low *p*CO_2_ may have occurred after six generations. Indeed, it is now widely recognized that evolutionary adaptation can occur over very short ecological timescales (Reznick and Ghalambor 2001; Stockwell et al. 2003). However, the low CO_2_ lines showed no significant changes in fecundity when exposed to elevated CO_2_ conditions (Fig. 4), suggesting that what we have observed could be irreversible effects of TGP persisting across generations. In fact, there is growing evidence to show that trans‐generational epigenetic effects can span across multiple (10+) generations (Jablonka and Raz 2009; Ho and Burggren 2010). In conclusion, the ambiguous nature of the results from our reciprocal transplant assay experiment prevents us from us pinpointing the underlying mechanism (i.e. phenotypic plasticity or evolutionary adaptation) which enabled worms to maintain fitness under low *p*CO_2_ conditions through to generation F7. Irrespective of the mechanism responsible, worms were able to maintain fitness levels across several generations.

##### TGP as a mechanism for facilitating evolutionary adaptation

If evolutionary adaptation to low *p*CO_2_ conditions had occurred, it was likely driven by TGP instead of natural selection. We base this conclusion on the lack of significant differences in juvenile survival between *p*CO_2_ treatments in any generation. Furthermore, all pairs produced viable offspring with no evidence of unfertilized eggs or delayed development. Together, these observations demonstrate that the strength of the selection environment was low. We predict that for species which are more resistant to rapid changes in *p*CO_2_, evolutionary adaptation may be driven primarily by TGP as opposed to natural selection.

Overall, *O. labronica* had a high capacity to exhibit phenotypic plasticity, both within and across generations. This is not surprising, as *O. labronica* typically occurs in highly heterogeneous environments, where phenotypic plasticity is generally evolutionary favoured (Ghalambor et al. 2007). Thus, it is reasonable to assume that the high levels of plasticity we observed in response to changes in *p*CO_2_ had evolved as a result of culturing worms under a highly variable pH regime for ~33 generations. In addition, many coastal environments experience substantial fluctuations in pH on a daily or seasonal basis that can be as large, or even greater, than the decrease in pH projected to occur over the next 50–100 years (Hofmann et al. 2011; Shaw et al. 2012; Melzner et al. 2013). Consequently, many coastal organisms may possess a high capacity to exhibit TGP. Indeed, a recent study showed that natural populations of the Atlantic silverside, *Menidia menidia,* were able to condition their offspring to seasonally acidifying environments (Murray et al. 2014). Such findings are important as TGP could buffer populations against the negative impacts of rapid changes in *p*CO_2_ allowing time for evolutionary adaptation to catch up (Chevin et al. 2010), or even facilitate evolutionary adaptation through processes such as the Baldwin effect and/or genetic assimilation (i.e. genetic accommodation) (Pigliucci et al. 2006; Crispo 2007).

##### No apparent costs associated with the multi‐generational responses

Plastic and evolutionary responses are often associated with costs and trade‐offs to life‐history traits and fitness (Hoffmann 1995; Angilletta et al. 2003). However, whilst we observed costs of within‐generation plasticity (i.e. reduced fecundity in generation F1 and F2), we detected no apparent costs for our observed multi‐generational responses. Although it is still possible that costs exist, potentially affecting traits such as maximum size, longevity and total life‐span reproductive output, all of which we were unable to determine with our experimental design. There is urgent need to identify the potential costs of TGP and evolutionary adaptation in response to changes in *p*CO_2_ and other climate change stressors (i.e. warming, hypoxia, combined stressors) if we are to more accurately predict whether current populations levels of marine metazoans will be able to persist under rapidly changing conditions. For example, if marine metazoans are able to persist at the cost of reduced body size (Gardner et al. 2011; Sheridan and Bickford 2011; Calosi et al. 2013; Garilli et al. 2015); body size‐dependent traits and processes (e.g. fecundity, competitive and predator‐prey interactions ‐ Peters 1983; Arendt 2007) and subsequently ecosystem functions may still be negatively impacted (Solan et al. 2004; Sheridan and Bickford 2011). Where feasible, future trans‐generational and multi‐generational studies should therefore characterize performance over entire life spans to better identify potential costs.

### Summary

Plastic and evolutionary responses are increasingly recognized as primary rescue mechanisms that could prevent species’ extinctions in the face of rapid climate change (Hoffmann and Sgrò 2011; Godbold and Calosi 2013; Gonzalez et al. 2013; Munday et al. 2013; Salinas et al. 2013; Sunday et al. 2014). Here, we show that a laboratory strain of *O. labronica* was able to rapidly (within two generations) restore its fitness levels, *via* TGP, and maintain these levels for a further four generations, after experiencing a change in *p*CO_2_. Regardless of whether TGP or evolutionary adaptation was the mechanism responsible for the results from our reciprocal transplant assay experiment, we provide evidence to suggest that marine metazoans may be capable of coping with drastic changes in pH conditions over multiple generations. Our study supports the idea that multi‐generational experiments are required to accurately predict how marine organisms will respond to climate change associated stressors (including *p*CO_2_) predicted to occur over the coming centuries (Munday et al. 2013; Sunday et al. 2014). Investigations that span across multiple generations will be valuable for the planning of socio‐economic and environmental buffering for the duration of the ‘detrimental’ phase (i.e. whilst fitness is reduced), while planning intervention actions that may preserve and/or speed up the ‘recovery’ phase. Finally, laboratory multi‐generational experiments could be used as a valuable conservation tool to select for strains tolerant to specific *p*CO_2_ conditions, with the scope to prevent the extinctions of keystone species in the face of rapid changes in *p*CO_2_ *via* the process of assisted evolution (Van Oppen et al. 2015).

### Data archiving statement

Life‐history, metabolic and water chemistry data have been deposited in the British Oceanographic Data Centre (http://www.bodc.ac.uk, doi:10.5285/22b54764‐2448‐1318‐e053‐6c86abc01ae1).

## Supporting information


**Table S1.** Mean values ±95 % CI for all traits measured for the marine polychaete *Ophryotrocha labronica* at elevated (grey rows) and low (white rows) *p* CO_2_ conditions across generations.

**Appendix S1**. Acclimation/selection to experimental conditions.
