# Supplementary material for: Multi‐generational responses of a marine polychaete to a rapid change in seawater p CO 2
Source: Evol Appl. 2015 Dec 18;9(9):1082–95. doi: 10.1111/eva.12344 (PMC5039322; doi:10.1111/eva.12344)
Supplement: Supplementary file 1 — Table S1. Mean values ±95 % CI for all traits measured for the marine polychaete Ophryotrocha labronica at elevated (grey rows) and low (white rows) p CO 2 conditions across generations. [file EVA-9-1082-s001.docx]

**Supplementary Table 1:** Mean values ± 95 % CI for all traits measured for the marine polychaete *Ophryotrocha labronica* at elevated (grey rows) and low (white rows) *p*CO_2_ conditions across generations. Capital and lower case letters represent significant differences (*P* < 0.05) between generations within the elevated and low *p*CO_2_ treatments respectively. Values in bold represent significant differences (*P* < 0.05) between *p*CO_2_ treatments within the same generation. Numbers of replicates are in parentheses.

| Trait | *p*CO_2_ treatment | Generation | | | | | | |
| --- | --- | --- | --- | --- | --- | --- | --- | --- |
|  |  | F1 | F2 | F3 | F4 | F5 | F6 | F7 |
| Juvenile growth rates (number of chaetigers d^-1^) | Elevated | 0.95 ± 0.05 ^A^  (40) | 1.26 ± 0.03 ^B^ (90) | 1.35 ± 0.04 ^C,D^  (85) | 1.39 ± 0.04 ^C,D^ (90) | 1.43 ± 0.04 ^D^ (90) | 1.34 ± 0.04 ^C^ (90) | 1.40 ± 0.03 ^C,D^ (90) |
|  | Low | 0.90 ± 0.04 ^a^ (40) | 1.25 ± 0.02 ^b^ (90) | 1.27 ± 0.04 ^b,c^ (90) | 1.42 ± 0.04 ^c,d^ (90) | 1.49 ± 0.05 ^d^ (80) | 1.34 ± 0.04 ^c^ (90) | 1.40 ± 0.04 ^c,d^  (90) |
| Juvenile survival (%) | Elevated | 85.0 ± 11.7 (8) | 82.5 ± 5.6 (18) | 80.9 ± 5.6 (17) | 85.8 ± 6.9 (18) | 88.6 ± 5.4 (18) | 87.5 ± 4.2 (18) | 82.2 ± 4.1 (18) |
|  | Low | 74.4 ± 16.4 (8) | 84.2 ± 5.2 (18) | 79.7 ± 8.3 (18) | 88.6 ± 5.2 (18) | 90.9 ± 4.4 (16) | 89.7 ± 3.8 (18) | 84.7 ± 2.9 (18) |
| Adult size (number of chaetigers) | Elevated | 14.35 ± 0.52 ^A^ (17) | 15.00 ± 0.35 ^A,B^ (17) | 15.61 ± 0.46 ^B^ (18) | 15.78 ± 0.24 ^B^ (18) | 15.83 ± 0.55 ^B^ (18) |  | 15.41 ± 0.29 ^B^ (17) |
|  | Low | 14.06 ± 0.44 ^A^ (17) | 15.72 ± 0.44 ^B^ (18) | 15.25 ± 0.32 ^B^ (16) | 16.00 ± 0.34 ^B^ (17) | 15.67 ± 0.36 ^B^ (18) |  | 15.39 ± 0.32 ^B^ (18) |
| Fecundity (number of eggs chaetiger^-1^) | Elevated | **6.70 ± 1.20 ^A^ (17)** | **8.12 ± 0.61 ^A,B^ (17)** | 9.92 ± 1.02 ^C^  (18) | 10.31 ± 1.15 ^C^ (18) | 9.32 ± 0.90 ^B,C^ (18) |  | 8.68 ± 0.71 ^B,C^  (17) |
|  | Low | **3.55 ± 0.44 ^a^  (17)** | **6.82 ± 0.63** ^b^ **(18)** | 9.78 ± 1.06 ^c^ (16) | 10.45 ± 1.18 ^c^ (17) | 9.60 ± 1.39 ^c^ (18) |  | 8.42 ± 0.66 ^c^  (18) |
| Egg volume (x 10^-3^ mm^3^) | Elevated | 0.60 ± 0.02 (170) | 0.60 ± 0.02 (170) | 0.61 ± 0.02 (180) | 0.60 ± 0.02 (180) | 0.62 ± 0.02 (180) |  | 0.62 ± 0.02 (170) |
|  | Low | 0.62 ± 0.02 (170) | 0.63 ± 0.02 (180) | 0.63 ± 0.02 (160) | 0.62 ± 0.02 (170) | 0.60 ± 0.02 (180) |  | 0.60 ± 0.02 (180) |
| Metabolic rates (μmol O2 h^-1^ STPD) | Elevated | 0.23 ± 0.03 (17) | 0.24 ± 0.04 (17) | 0.27 ± 0.03 (18) | 0.27 ± 0.03 (18) | 0.30 ± 0.04 (18) |  | 0.28 ± 0.05 (9) |
|  |  |  |  |  |  |  |  |  |
|  | Low | 0.22 ± 0.02 ^a^  (17) | 0.29 ± 0.06 ^a,b^  (18) | 0.27 ± 0.02 ^b^  (16) | 0.26 ± 0.02 ^a,b^  (17) | 0.28 ± 0.03 ^b^ (18) |  | 0.29 ± 0.06 ^a,b^ (9) |
