# Supplementary material for: Multi‐generational responses of a marine polychaete to a rapid change in seawater p CO 2
Source: Evol Appl. 2015 Dec 18;9(9):1082–95. doi: 10.1111/eva.12344 (PMC5039322; doi:10.1111/eva.12344)
Supplement: Supplementary file 2 — Appendix S1. Acclimation/selection to experimental conditions. [file EVA-9-1082-s002.docx]

**Supporting information**

**Acclimation/selection to experimental conditions**

Our significant ‘generation’ effects strongly support the idea that acclimation and/or selection to the experimental conditions had occurred. The significant ‘generation’ effects we observed (juvenile growth rates, adult size and fecundity) were likely the product of artificial selection imposed by pairing individuals which reached sexual maturity first, and were therefore faster growers. Faster growth rates would result in adults reaching a larger size earlier, and as a result potentially having increased fecundity, compared to previous generations, due to the positive correlation between body size and fecundity with *O. labronica* (Berglund, 1991). In addition, the change in temperature between laboratory culture and experimental conditions (20 °C *vs.* 27 °C) could have also played a role in our observed ‘generation’ effects. Parker et al. (2011, 2012) showed that selectively bred lines of oyster for faster growth were more resilient to OA compared to wild populations. This study reports similar results. Although fecundity was significantly greater in the elevated-elevated *p*CO_2_ transplant compared to the elevated-low *p*CO_2_ transplant (generation F7), the difference was not as great compared to generation F1. Similarly to Parker et al. (2011, 2012), the significantly higher juvenile growth rates in generation F7, as a either a result of acclimation and/or selection to the experimental conditions, could be the underlying mechanism for the observed increase in resilience to changes in *p*CO_2_ levels compared to generation F1.

References

Berglund, A. 1991. To change or not to change sex: A comparison between two *Ophryotrocha* species (Polychaeta). Evolutionary Ecology **5**:128-135.

Parker, L.M., O. M. Ross and W. A. O’Connor 2011. Populations of the Sydney rock oyster, *Saccostrea glomerata*, vary in response to ocean acidification. Marine Biology **158**:689-697.

Parker, L.M., P. M. Ross, W. A. O’Connor, L. Borysko, D. A. Raftos and H-O Pörtner 2012. Adult exposure influences offspring response to ocean acidification in oysters. Global Change Biology **18**:82-92.
